# Role of the *luxS* gene in bacteriocin biosynthesis by *Lactobacillus plantarum* KLDS1.0391: A proteomic analysis

**DOI:** 10.1038/s41598-017-13231-4

**Published:** 2017-10-24

**Authors:** Fang-Fang Jia, Xue-Hui Pang, De-Quan Zhu, Zong-Tao Zhu, Si-Rui Sun, Xiang-Chen Meng

**Affiliations:** 1 Key Laboratory of Dairy Science, Ministry of Education, Northeast Agricultural University, Harbin, 150030 China; 2 Synergetic Innovation Center of Food Safety and Nutrition, Northeast Agricultural University, Harbin, 150030 China; 3 College of Life Sciences, Jiamusi University, Jiamusi, 154007 China

## Abstract

Certain probiotic species of lactic acid bacteria, especially *Lactobacillus plantarum*, regulate bacteriocin synthesis through quorum sensing (QS) systems. In this study, we aimed to investigate the *luxS*-mediated molecular mechanisms of QS during bacteriocin synthesis by *L. plantarum* KLDS1.0391. In the absence of *luxS*, the ‘spot-on-the-lawn’ method showed that the bacteriocin production by *L. plantarum* KLDS1.0391 significantly decreased upon co-cultivation with *L. helveticus* KLDS1.9207 (*P* < 0.01) but did not change significantly when mono-cultivated. Furthermore, liquid chromatography-electrospray ionization tandem mass spectrometry analysis showed that, as a response to *luxS* deletion, *L. plantarum* KLDS1.0391 altered the expression level of proteins involved in carbohydrate metabolism, amino acid metabolism, fatty acid synthesis and metabolism, and the two-component regulatory system. In particular, the sensor histidine kinase AgrC (from the two-component system, LytTR family) was expressed differently between the *luxS* mutant and the wild-type strain during co-cultivation, whereas no significant differences in proteins related to bacteriocin biosynthesis were found upon mono-cultivation. In summary, we found that the production of bacteriocin was regulated by carbohydrate metabolism, amino acid metabolism, fatty acid synthesis and metabolism, and the two-component regulatory system. Furthermore, our results demonstrate the role of *luxS*-mediated molecular mechanisms in bacteriocin production.

## Introduction

Lactic acid bacteria (LAB) produce antimicrobial metabolites and have been traditionally used as starter cultures for different fermented foods, medicine, and feed. The production of metabolites such as organic acids, ethanol, hydrogen peroxide, and diacetyl is associated with the preservative and inhibitory effects of a few bacterial strains^1^. The preservative effect of many LAB is likely due in part to their bacteriocin production, which provides an advantage to producers in competing with other bacteria sharing the same ecological niche^2,3^. For example, *Lactobacillus plantarum* constitutes a flexible and versatile facultative heterofermentative LAB found in food environments such as vegetables, meat, aquatics, dairy products, and grape must, as well as in the gastrointestinal tracts of humans and animals. Accordingly, to enable effective adaptation to changeable environmental conditions (e.g. co-cultivation with other bacteria, pH, and heat), *L. plantarum* requires quorum sensing (QS) systems to detect specific environmental signals^4^.

QS, in which gene transcription is regulated in response to a change in cell density, is mediated by direct cell-cell contact or by the synthesis, release, and detection of small signalling molecules^5^. The QS system comprises two components: the first consists of signalling molecules, which are referred to as autoinducers (AIs, including AI-1 and AI-2) or AI peptides (AIP); the second is the two-component regulatory system, which comprises the membrane-located histidine protein kinase that monitors one or more environmental factors, as well as the cytoplasmic response regulator that modulates the expression of specific genes. Through adopting co-culture conditions or by constructing a two-component or AI-2/*luxS* mutant strain, previous studies^6,7^ have demonstrated that bacteriocin production is regulated via the QS pathway. Specifically, the induction of bacteriocin production by co-culture is widespread among bacteriocin-producing *L. plantarum* strains^8^. In particular, AI-2, which constitutes a by-product of the activated methyl cycle by which S-adenosylmethionine (SAM) is recycled, might play a role in the synthesis of bacteriocin^9^. AI-2 is formed by the catalysis of S-ribosylhomocysteine (SRH) via the LuxS enzyme, where SRH is the product of detoxification of S-adenosylhomocysteine, a demethylated product of SAM, by the enzyme Pfs^9^. The involvement of LuxS in the production of AI-2 is often found in *Firmicutes* and more particularly in *Lactobacillus* ^10^. Although the role of LuxS in the AI-2 biosynthetic pathway is consistent across different bacterial species, as summarized by Pereira *et al*.^9^, the AI-2 signal export and reception/transduction pathways in *Lactobacillus* spp., or closely related genera, have not yet been elucidated^11^. In addition to genetic tools, proteomic studies on QS, particularly under stressful conditions, such as co-cultivation with certain bacteria^12^, and presence of a *luxS* mutation^13^, might provide a more comprehensive view of the bacteriocin production mechanisms.

*L. plantarum* KLDS1.0391 was isolated from ‘jiaoke’, a traditional, naturally fermented cream from Inner Mongolia in China. The bacteriocin produced by this strain, plantaricin MG, offers the advantages of a broad inhibitory spectrum, wide pH tolerance, and heat stability, but is produced at lower levels than nisin produced by the commercial strain *L. lactis* AL2^14,15^. Furthermore, we found that the bacteriocin production by *L. plantarum* KLDS1.0391 was markedly increased (*P* < 0.01) when co-cultivated with *L. helveticus* KLDS1.9207^16^, a strain that does not produce bacteriocins. In addition, *L. plantarum* KLDS1.0391 possesses an AI-2-mediated two-component system^16^, whereas *L. helveticus* KLDS1.9207 does not. Given that AI-2 might play a role in the synthesis of bacteriocins, we deduced that the *luxS* gene might be associated with the biosynthesis step of bacteriocin production. Moreover, bacteriocin production by *L. plantarum* KLDS1.0391 was markedly influenced (*P* < 0.05) by the co-cultivation conditions^15^. However, whether the effect of *luxS* on bacteriocin production is affected by the selective culture conditions remains to be determined.

Therefore, in our previous research, we constructed a *luxS* mutant strain of *L. plantarum* KLDS1.0391 by homologous recombination (manuscript submitted, under review) to illustrate the effect of *luxS* on bacteriocin production in mono-cultivation and co-cultivation with *L. helveticus* KLDS1.9207. In the present study, we further aimed to investigate *luxS*-mediated molecular mechanisms in the bacteriocin synthesis by *L. plantarum* KLDS1.0391 upon co-cultivation with *L. helveticus* KLDS1.9207 and during mono-cultivation, using a label-free quantitative shotgun proteomics strategy.

## Results

### Comparison of live cell number and bacteriocin production between *luxS* mutant and the wild-type strain in mono- and co-cultivation with *L. helveticus* KLDS1.9207

**Figure 1:**
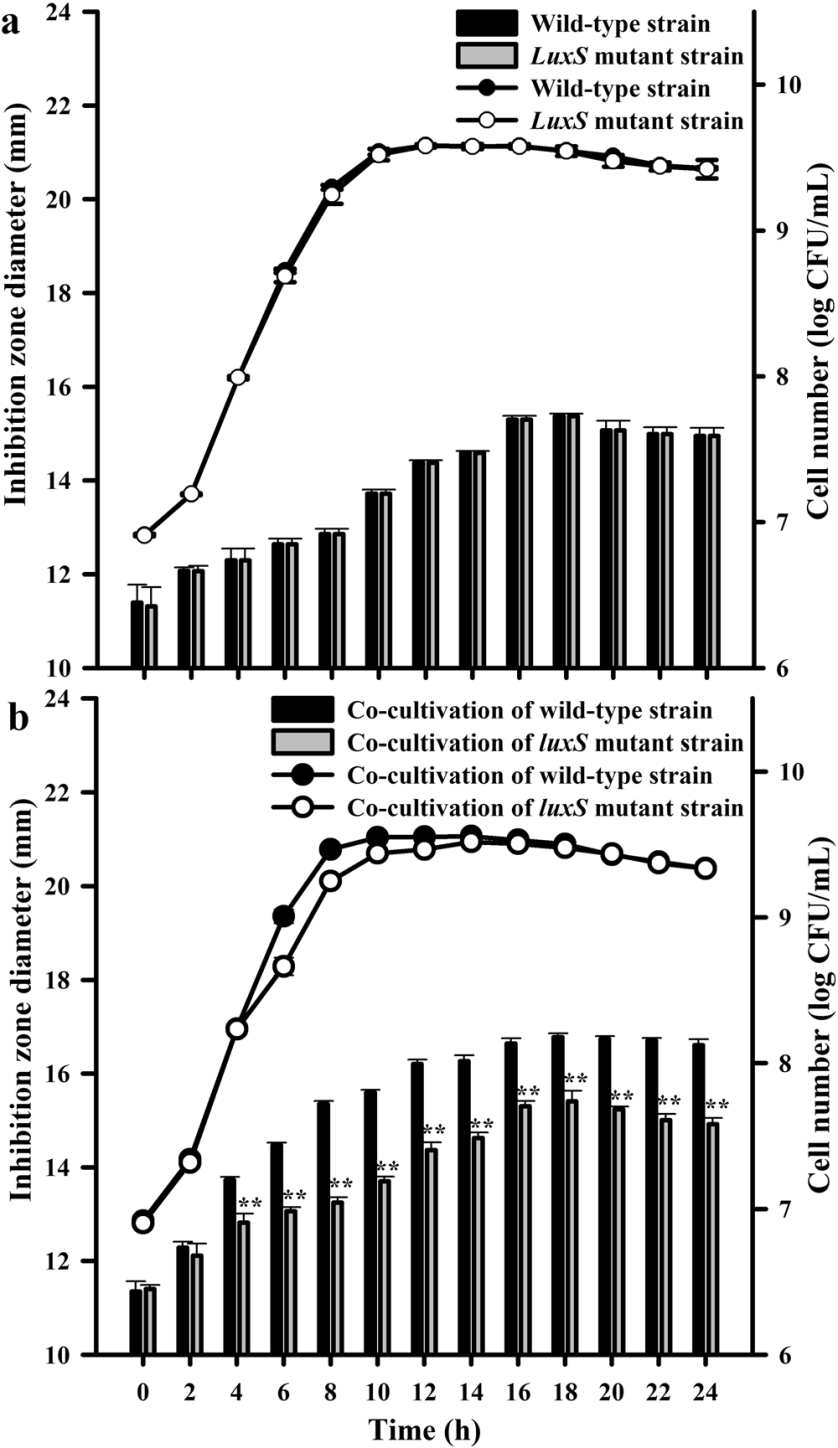
Cell number (, ) and inhibitory activity (, ) of wild-type and *luxS* mutant strains in mono-cultivation (**a)** and co-cultivation with *L. helveticus* KLDS1.9207 (**b**). Cell number and inhibition zone diameter (inhibitory activity) are expressed as the means ± standard deviation (SD; n = 3). **Statistically significant difference between wild-type strain and *luxS* mutant strain (*P* < 0.01).

The live cell numbers and inhibition zone diameters of the *luxS* mutant and wild-type strains in mono-cultivation (a) and in co-cultivation (b) with *L. helveticus* KLDS1.9207 are shown in Fig. 1. The live cell number of the *luxS* mutant strain compared to that of the wild-type strain in mono-cultivation was not markedly changed (*P* > 0.05) but was significantly lower than that of the wild-type strain upon co-cultivation with *L. helveticus* KLDS1.9207 during a growth period of 6–12 h (*P* < 0.01). The antibacterial activity of the *luxS* mutant strain was significantly decreased (*P* < 0.01) compared with that of the wild-type strain in co-cultivation with *L. helveticus* KLDS1.9207 during growth for 4–24 h; however, the antibacterial activity showed little change during mono-cultivation.

### Differentially expressed proteins between the wild-type and *luxS* mutant strains in mono- and co-cultivation with *L. helveticus* KLDS1.9207

In accordance with the selection criteria of ratio >±2 and *P* value < 0.05, we identified 108 differentially expressed proteins (Table 1) from the mono-cultivation group and 49 differentially expressed proteins (Table 2) from the co-cultivation group. The 108 proteins from the mono-cultivation group included 39 significantly differently expressed proteins (26 and 13 proteins with significant down- or upregulation, respectively) and 69 proteins for which the expression was below the detection limit of mass spectrometry (MS). The 49 proteins from the co-cultivation group included 13 significantly differentially expressed proteins (2 and 11 proteins with significant down- or upregulation, respectively) and 36 proteins below the MS detection limit.

**Table 1:** Differentially expressed proteins between the *luxS* mutant and the wild-type strain in mono-cultivation.

NO.	Protein ID	Map Name	Sequence description	Quantitative change and significance	
				A/B^a^	*P* value
1	A0A0R2GFJ4	PTS-Bgl-EIIA, bglF, bglP	PTS system trehalose-specific IIB component	0.477850866	4.015
2	A0A0R2G9N6	DLAT, aceF, pdhC	Dihydrolipoamide acetyltransferase component of pyruvate dehydrogenase complex	0.334091875	6.857
3	A0A166KZ80	E2.4.1.8, mapA	Maltose phosphorylase	0.124908833	0.000
4	A0A0G9FF05	msmX, msmK, malK, sugC, ggtA, msiK	Maltose maltodextrin transport ATP-binding	0.032826035	0.000
5	A0A166HX81		Promiscuous sugar phosphatase haloaciddehalogenase-like phosphatase family	0.458219978	0.001
6	A0A162HJ67	pgmB	Beta-phosphoglucomutase	0.224240296	0.001
7	A0A0R1UMU3	PDHA, pdhA	Pyruvate dehydrogenase E1 component alpha subunit	0.30363946	0.002
8	A0A0G9FDH3	DLD, lpd, pdhD	Dihydrolipoamide dehydrogenase of pyruvate dehydrogenase complex	0.335633652	0.002
9	A0A0R1UYF1	PTS-Cel-EIIB, celA, chbB	PTS system cellobiose-specific IIB component	0.313399828	0.002
10	A0A0G9FEW8	cycB, ganO	Sugar ABC transporter substrate-binding	0.095777705	0.002
11	A0A166LCG7		Oxidoreductase aldo keto reductase family	0.22210812	0.002
12	A0A166J2F1	rbsK, RBKS	Ribokinase	0.399403783	0.002
13	A0A0P7HSH4	hprK, ptsK	HPr kinase phosphorylase	0.389065012	0.004
14	A0A166K0Z7	PDHB, pdhB	Pyruvate dehydrogenase E1 component beta subunit	0.327629226	0.004
15	A0A0G9F747	PTS-Man-EIIC, manY	PTS system mannose-specific IIC component	0.443786982	0.004
16	D7V9C7	malY, malT	Sugar transporter	0.145274287	0.007
17	A0A151G230	galM, GALM	Galactose mutarotase	0.328294689	0.018
18	A0A0P7HQL7		NADH oxidase	0.374619026	0.019
19	Q88WV2	nrdR	Transcriptional regulator	0.391799787	0.025
20	P59407	E4.1.3.3, nanA, NPL	N-acetylneuraminate lyase	0.169766187	0.027
21	A0A0G9FD31	E2.4.1.8, mapA	Maltose phosphorylase	0.127697815	0.030
22	A0A0R2GC45	alsD, budA, aldC	Alpha-acetolactate decarboxylase	0.42085536	0.046
23	A0A0G9F7H9		Malolactic regulator	0.423614866	0.046
24	D7V885	ackA	Acetate kinase	0.485433255	0.046
25	D7V7S0	thiM	Hydroxyethylthiazole kinase	0.415559565	0.046
26	A0A0G9GMV5	GSR, gor	Glutathione reductase	0.399381865	0.050
27	D7V8Y5	glk	Glucokinase	11.67055987	0.000
28	A0A166LM67	E3.2.1.17	Cell wall hydrolase	2.178588789	0.001
29	A0A166LGI2		Glycoside hydrolase family 25	2.239080765	0.005
30	A0A151G2W4	PTS-Nag-EIIC, nagE	PTS N-acetylglucosamine transporter subunit IIABC	2.406888508	0.007
31	A0A165US72	E1.17.4.1 A, nrdA, nrdE	Ribonucleotide reductase of class Ib alpha subunit	2.079065281	0.007
32	A0A0G9GSZ0	pgmB	Beta-phosphoglucomutase	2.059377081	0.008
33	A0A0N8I4I6		Alcohol dehydrogenase	3.131477189	0.012
34	Q88YZ4	fabH	3-oxoacyl-(acyl-carrier-) synthase KASIII	2.254202031	0.014
35	A0A0G9FGA4		Diadenosine tetraphosphatase and related serine threonine phosphatase	2.401060831	0.016
36	A0A0P7HQH4		Hypothetical protein	3.610968428	0.018
37	A0A166H1G4	K06904	Phage capsid protein	2.019174041	0.019
38	D7VEU7	K06889	Hydrolase of the alpha beta superfamily	2.462973125	0.020
39	A0A0G9FH00		Multispecies: hypothetical protein	2.438489371	0.023
40	Q88T16	E5.2.1.8	Foldase precursor		
41	Q88V03	ruvB	Holliday junction DNA helicase		
42	Q88V79	mraY	Phospho-N-acetylmuramoyl-pentapeptide-transferase		
43	Q88WJ2	trmD	tRNA -methyltransferase		
44	Q88WP5	miaA, TRIT1	tRNA dimethylallyltransferase		
45	Q88XV1	ecfA2	ATPase component of ral energizing module of ECF transporter		
46	Q88ZU5	serC, PSAT1	Phosphoserine aminotransferase		
47	A0A059UCU6	ganP	Maltose maltodextrin ABC transporter permease		
48	A0A0G9F7Q4	ABC.CD.A	ABC transporter ATP-binding protein		
49	A0A0G9F9N1	rluD	RNA pseudouridine synthase		
50	A0A0G9F9S7		HAD family hydrolase		
51	A0A0G9F9Y3		Nudix-related transcriptional regulator		
52	A0A0G9FAX4		HAD family hydrolase		
53	A0A0G9FBB9		Hypothetical protein		
54	A0A0G9FCP4		Cell surface protein		
55	A0A0G9FHS8		Negative regulator of proteolysis		
56	A0A0G9GIU3	GSP13	General stress protein		
57	A0A0G9GQE3	K06910	Phosphatidylethanolamine-binding protein		
58	A0A0G9GQZ7		Multispecies: hypothetical protein		
59	A0A0L7Y046		Transcription regulator (contains diacylglycerol kinase catalytic domain)		
60	A0A0L7Y0D5		Hypothetical protein		
61	A0A0L7Y739		Acyl- hydrolase		
62	A0A0M0CEA0		Regulator		
63	A0A0M0CFS2		Damage-inducible J		
64	A0A0M0CG41	E1.2.3.3, poxL	Pyruvate oxidase		
65	A0A0M0CHM2	treC	Trehalose-6-phosphate hydrolase		
66	A0A0M4CWX9		Methionine–tRNA ligase		
67	A0A0P7GJ96		Hypothetical protein		
68	A0A0P7HFF8		DUF2273 domain-containing		
69	A0A0P7HGY1	ABC-2.P	ABC transporter permease		
70	A0A0P7HHH5		Hypothetical protein		
71	A0A0P7HNH7		Hypothetical cytosolic		
72	A0A0P7HSW4		ISSag6 transposase		
73	A0A0P7IQD5		Stress response regulator Gls24		
74	A0A0R1UP09	iunH	Inosine-uridine preferring nucleoside hydrolase		
75	A0A0R1USD0	coaE	Dephospho- kinase		
76	A0A0R1V037		ORF00007-like (plasmid)		
77	A0A0R1V1M0	ribT	Riboflavin biosynthesis acetyltransferase family		
78	A0A0R1V308		Extracellular		
79	A0A0R1V7I4		Conjugal transfer		
80	A0A0R2G5K4		Lipoprotein		
81	A0A0R2G8W3	rlmA1	Ribosomal RNA large subunit methyltransferase A		
82	A0A0R2GD86	E1.2.3.3, poxL	Pyruvate oxidase		
83	A0A0R2GG38		TPR repeat-containing		
84	A0A0R2GH14		Isochorismatase		
85	A0A151G1C3		Transcription regulator		
86	A0A151G5I5		Membrane (plasmid)		
87	A0A162EN38	virD4, lvhD4	Conjugal transfer		
88	A0A162GM58		Multispecies: hypothetical protein		
89	A0A162GZ91		Conjugal transfer		
90	A0A165DXD9	phoR	Phosphate regulon sensor		
91	A0A165EXC6		Hypothetical protein		
92	A0A165VBP4	fabK	2-nitropropane dioxygenase		
93	A0A165X1Y3		D-3-phosphoglycerate dehydrogenase		
94	A0A165ZPF4		Cell surface protein		
95	A0A166FZ63		Plasmid replication initiation		
96	A0A166P0P2		Transposase		
97	C3U0I3		rRNA adenine N-6-methyltransferase		
98	D7VDC6		Lipoprotein		
99	D7VEF6		DNA double-strand break repair Rad50 ATPase		
100	T5JG80	K09963	Outer surface protein		
101	T5JJD7	ABC.PE.S	Peptide ABC transporter substrate-binding		
102	T5JNS0		Rrf2 family transcriptional regulator		
103	T5JPM7		Membrane anchor connecting 2 with cell-division Z-ring		
104	T5JTG7		Biphenyl-2 3-diol 1 2-dioxygenase III-related		
105	T5JY38	ispE	4-diphosphocytidyl-2-C-methyl-D-erythritolkinase		
106	T5K0G6		Hypothetical protein		
107	U2XGM5	priA	Primosomal protein N		
108	U2XSX3		Putative ABC transporter, permease protein		

^a^A: *LuxS* mutant strain; B: Wild-type strain.

**Table 2:** Differentially expressed proteins between the *luxS* mutant and the wild-type strain in co-cultivation with *L. helveticus* KLDS1.9207.

NO.	Sequence name	Map Name	Sequence description	Quantitative change and significance	
				C/D^b^	*P* value
1	P77887	pyrDI	Dihydroorotate dehydrogenase catalytic subunit	2.760886385	0.031
2	A0A0G9FAP4		Transcriptional regulator family	2.945902345	0.003
3	A0A0G9FCW2		GNAT family acetyltransferase	2.070695848	0.030
4	A0A0L7XZQ3		Gamma-D-glutamyl-meso-diaminopimelate peptidase	2.747063118	0.036
5	A0A0R1UXL5	E4.1.1.15	Glutamate decarboxylase	2.146220812	0.039
6	A0A0R1VEW0		Transcriptional regulator	0.45197274	0.028
7	A0A0R2GIZ8		Uncharacterized protein	2.038790183	0.003
8	D7VFU5	htpX	Heat shock	0.427200477	0.001
9	M4KFL2		Acyltransferase	2.708140733	0.004
10	U2W2U5		Multispecies: hypothetical protein	2.282826367	0.016
11	U2W7H2		D-lactate dehydrogenase	2.019712705	0.045
12	U2WKG8	prsA	Peptidylprolyl isomerase	2.070595076	0.042
13	U2WLF8		Nucleoside 2-deoxyribosyltransferase	2.311437674	0.013
14	C6VLJ0	accD	Acetyl- carboxyl transferase		
15	Q88VX7	clpB	ATP-dependent chaperone		
16	Q88WT1	agrC, blpH, fsrC	UPF0348 lp_1534		
17	A0A0G9F856		Histidine kinase		
18	A0A0G9F9S7		HAD family hydrolase		
19	A0A0G9FBJ9		Oxidoreductase aldo keto reductase family		
20	A0A0G9FCA3		Dimeric dUTPase		
21	A0A0G9FE10	recX	Recombinase		
22	A0A0G9FGT8	fabG	3-oxoacyl-(acyl-carrier) reductase		
23	A0A0G9GJI0	nrdG	Ribonucleoside-triphosphate reductase activating		
24	A0A0G9GKX1		GNAT family acetyltransferase		
25	A0A0G9GR36		Transcriptional regulator		
26	A0A0G9GTJ1		Transcriptional regulator		
27	A0A0G9GU14	ABC.CD.P	ABC transporter permease		
28	A0A0G9GU74	murF	UDP-N-acetylmuramoyl-tripeptide–D-alanyl-D-alanine ligase		
29	A0A0G9GUG9	GSR, gor	Glutathione reductase		
30	A0A0L7XZK6	PTS-Gut-EIIA, srlB	PTS system IIA component		
31	A0A0M0CGA8		Diadenosine tetraphosphate hydrolase		
32	A0A0M0CHX3	rsmC	Ribosomal RNA small subunit methyltransferase C		
33	A0A0P7H5T1	relA	GTP pyrophosphokinase		
34	A0A0R1UDH2		DUF2179 domain-containing		
35	A0A0R1UU28	NARS, asnS	Asparaginyl-tRNA synthetase		
36	A0A0R1V3K0		Trehalose operon transcriptional repressor		
37	A0A0R1V4C9		Branched-chain amino acid ABC transporter		
38	A0A0R1V4X3	patA	D-lactate dehydrogenase		
39	A0A0R2G4A4		Transcription regulator		
40	A0A151G5A1		Hypothetical protein		
41	A0A151G5L5		Lantibiotic epidermin biosynthesis		
42	A0A162E1B4		Nucleoside 2-deoxyribosyltransferase		
43	A0A165P9S6	ydjE	Niacin transporter		
44	D7V8R3	K06878	Phenylalanyl-tRNA synthetase domain		
45	T5JD50	gshA	Bifunctional glutamate–cysteine ligase		
46	T5JD81		Glutamine amidotransferase		
47	T5JHA9	K07009	DegV family EDD domain-containing protein		
48	T5JPL2	ftsZ	Cell division protein FtsZ		
49	U2WPC9		Lactate oxidase		

^b^C: Co-cultivation of the *luxS* mutant strain with *L. helveticus* KLDS1.9207; D: Co-cultivation of the wild-type strain with *L. helveticus* KLDS1.9207.

**Figure 2:**
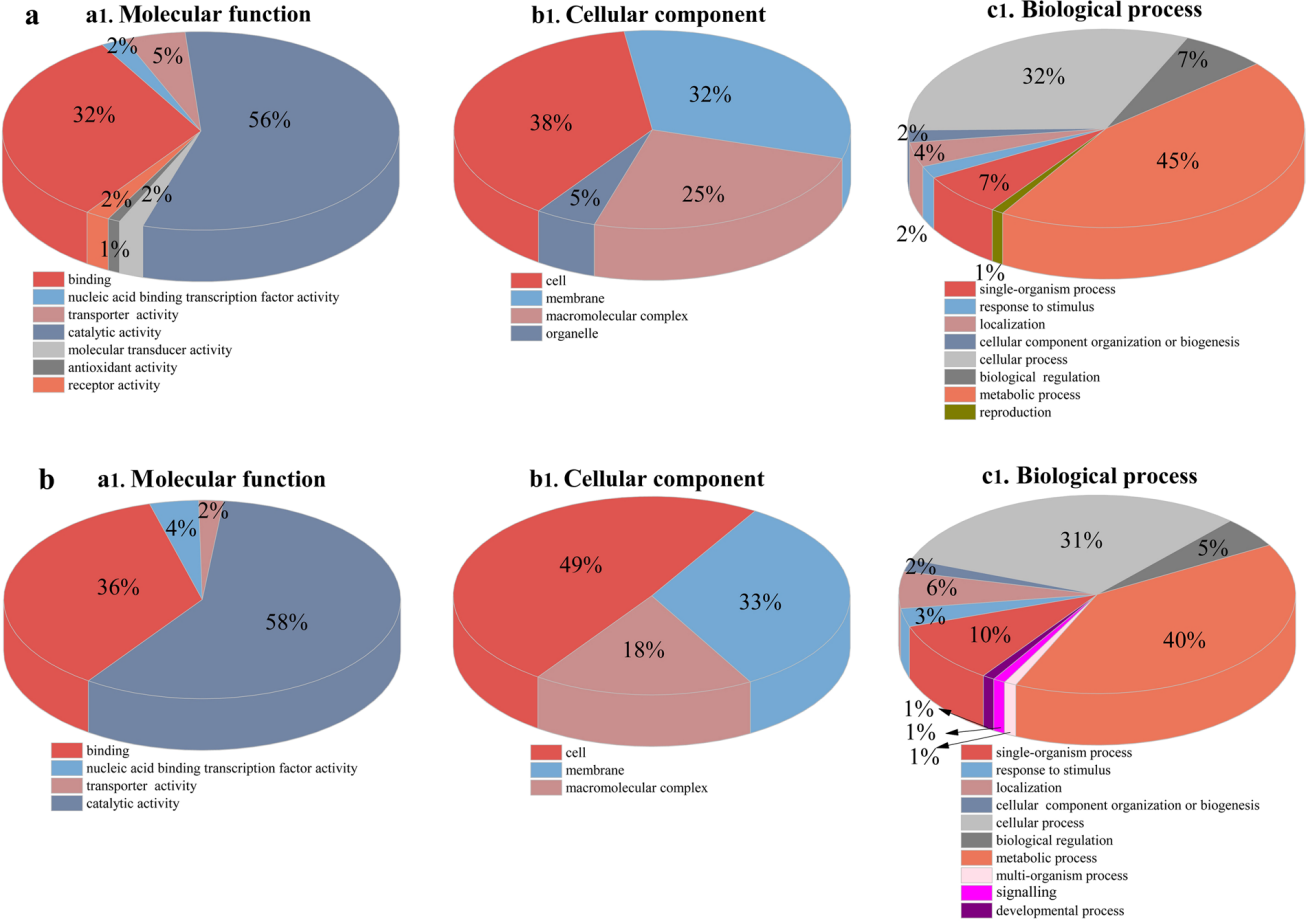
Map of gene ontology (GO) annotation. Classifications of all altered proteins in mono-cultivation (**a**) and co-cultivation (**b**), based on molecular function (**a1**), subcellular localization (**b1**), and biological process (**c1**).

To characterize the set of proteins with decreased or increased expression for biological interpretation, gene ontology (GO) analysis was performed. The results of GO analysis showed that all identified differentially expressed proteins have different molecular functions and are involved in different cellular components; they also participate in different biological processes in the cell (Fig. 2). For the molecular function categories, all differentially expressed proteins were classified into seven functional groups in mono-cultivation but only into four groups in co-cultivation. The majority of the differentially expressed proteins in both mono- and co-cultivation conditions have catalytic activity or act as binding proteins (Fig. 2a[a1] and b[a1]). The cellular component ontology of proteins refers to the location in the cell where proteins are active^17^. Among these altered proteins, the majority in both groups are located in the cell, membrane, and macromolecular complexes, whereas differentially expressed proteins in organelles were only found in mono-cultivation (Fig. 2a[b1] and b[b1]). The altered proteins participate in a wide range of biological processes, such as metabolic, cellular, and single-organism processes (Fig. 2a[c1] and b[c1]).

**Figure 3:**
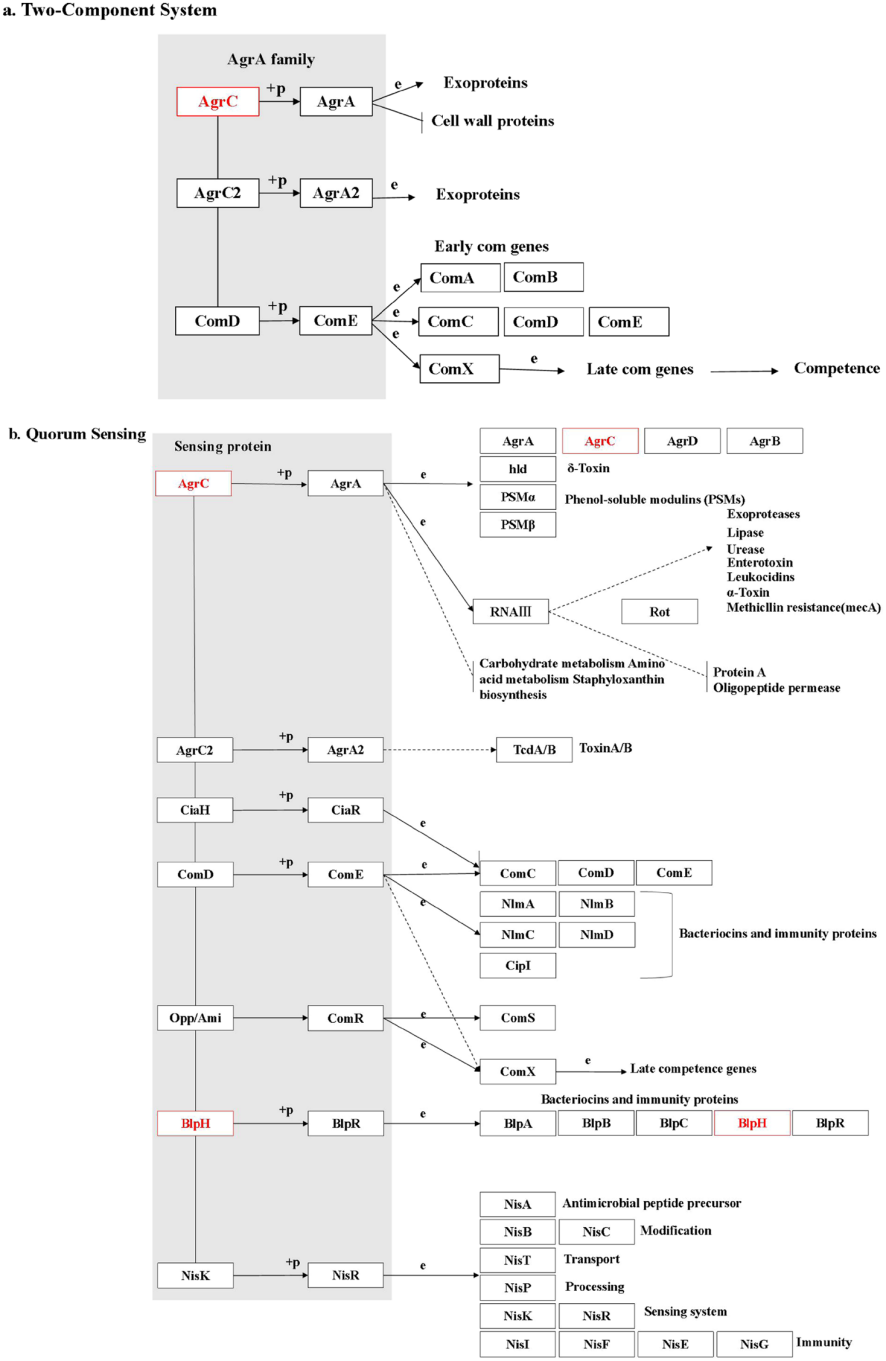
Kyoto Encyclopedia of Genes and Genomes (KEGG) pathway for biosynthesis of bacteriocin [(**a**) two-component system, (**b**) quorum sensing]. Red represents proteins with decreased expression in *L. plantarum* KLDS1.0391 co-cultivated with *L. helveticus* KLDS1.9207 on the graphic pathway map when *luxS* was deleted. Objects:  gene product, mostly protein but including RNA; Arrows:  molecular interaction or relation; Protein-protein interactions:  phosphorylation,  activation,  inhibition,  indirect effect,  binding/association,  complex; Gene expression relation: expression,  indirect effect.

**Figure 4:**
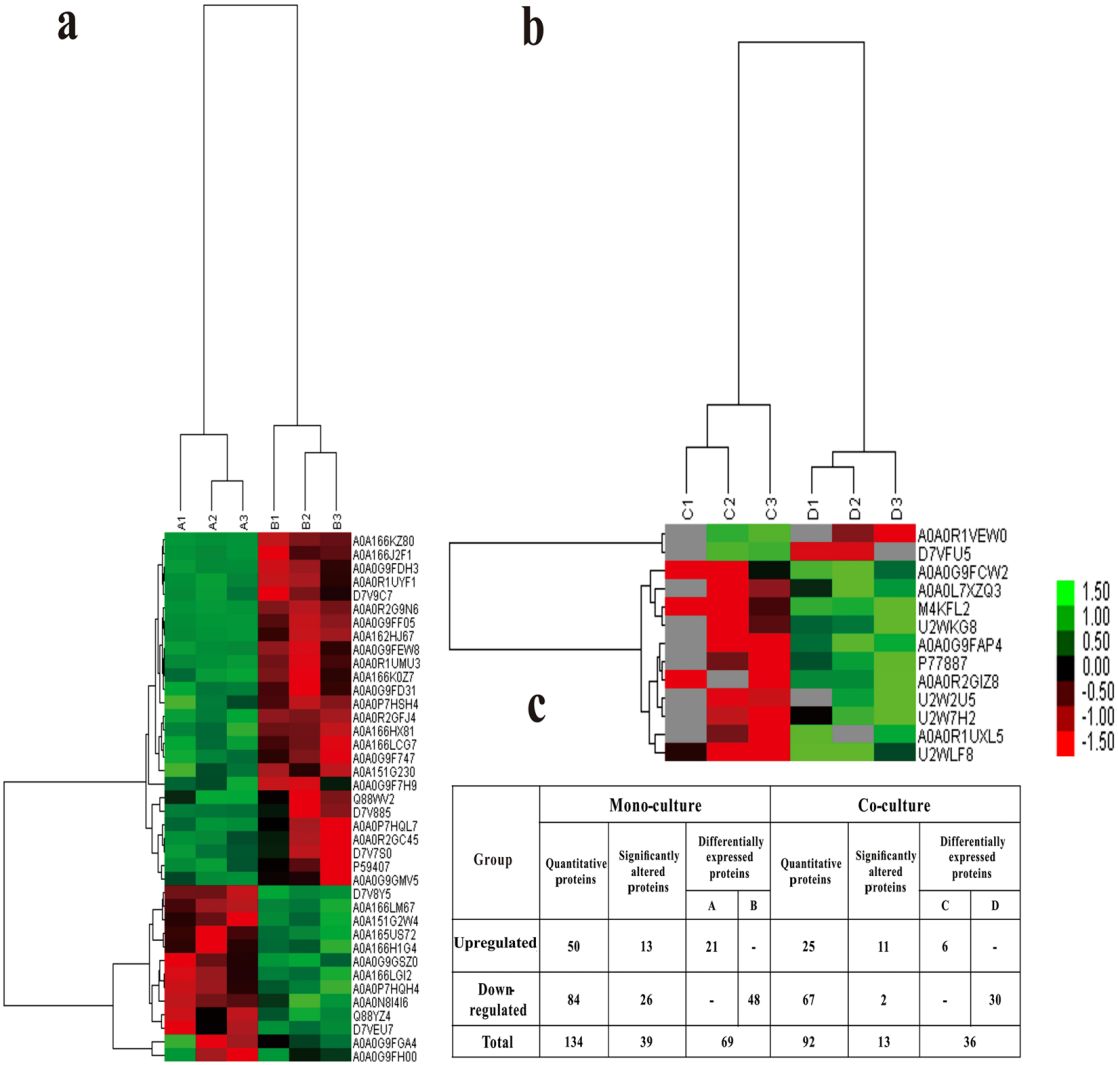
Heatmap of obviously altered proteins in mono-cultivation (**a**) and co-cultivation (**b**). A1, A2, A3- *L. plantarum* KLDS1.0391 *luxS* mutant strain; B1, B2, B3- *L. plantarum* KLDS1.0391 wild-type strain; C1, C2, C3- KLDS1.0391 *luxS* mutant strain co-cultivated with *L. helveticus* KLDS1.9207; D1, D2, D3- KLDS1.0391 wild-type strain co-cultivated with *L. helveticus* KLDS1.9207. Up- and downregulated proteins are indicated in shades of green (increased) and red (decreased), respectively. (**c**) Number of differential proteins. ‘−’ indicates that protein expression was lower than the detection limit of MS.

In addition, the Kyoto Encyclopedia of Genes and Genomes (KEGG) pathway annotation for the co- (Fig. 3) and mono-cultivation groups (Supplementary Fig. S3) was analysed to delineate the effects of *luxS* on the networks of related molecules in bacteriocin biosynthesis. Figure 3 shows that the expression of the sensor histidine kinases ArgC and BlpH (two-component system) belonging to the LytTR family changed significantly (*P* < 0.01) upon co-cultivation. The LytTR domain is a DNA-binding domain that functions to activate or inhibit the transcription of a particular gene^18^; thus, it may activate the transcription of the gene encoding bacteriocin^6^. In contrast, the expression of proteins associated with bacteriocin synthesis involved in the QS and two-component system pathways did not change during mono-cultivation (Table 1), although the expression of ABC.PE.S protein, which is related to virulence or biofilm formation and is involved in QS and two-component system pathways, was altered in mono-cultivation (Supplementary Fig. S3). Clustering analysis showed high repeatability among three biological replicates, regardless of the cultivation group. Moreover, the protein expression between *L. plantarum* KLDS1.0391 wild-type and *luxS* mutant strains obviously differed in each cultivation group (Fig. 4a and b). In addition, a larger number of altered proteins were identified in the mono-cultivation group than in the co-cultivation group when the *luxS* gene was deleted (Fig. 4c).

### Validation of the identified proteins

**Figure 5:**
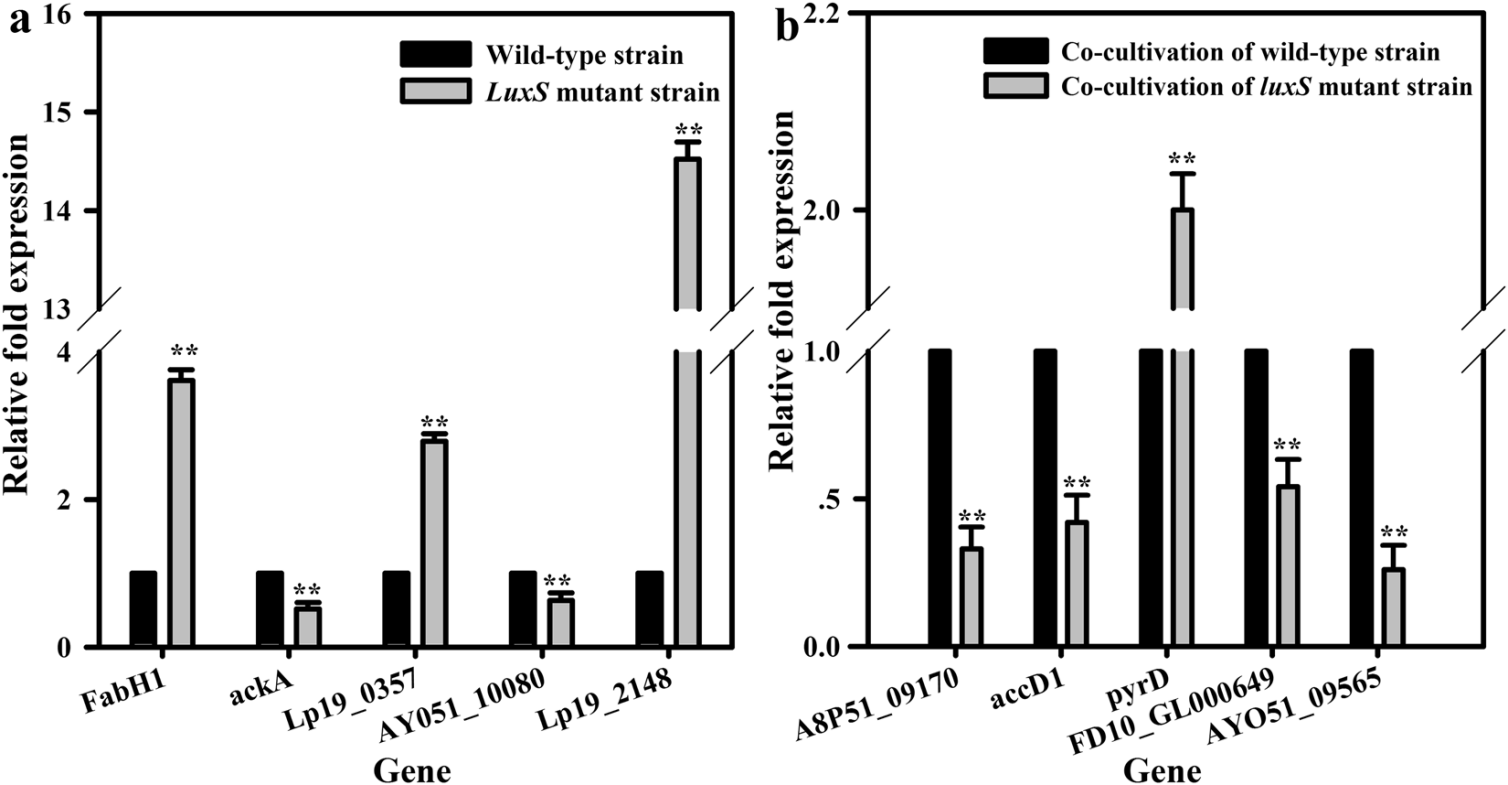
Quantitative real-time reverse transcription PCR (qRT-PCR) analysis of gene expression of altered proteins in mono-cultivation of *L. plantarum* KLDS1.0391 [(**a**) including five altered proteins] and co-cultivation of *L. plantarum* KLDS1.0391 with *L. helveticus* KLDS1.9207 [(**b**) including five altered proteins] upon *luxS* knockout. **Statistically significant difference between *L. plantarum* KLDS1.0391 wild-type strain and *luxS* mutant strain (*P* < 0.01).

We chose 10 proteins from among those differentially expressed in mono-cultivation (i.e. FabH1, ackA, Lp19_0357, AY051_10080, and Lp19_2148) and co-cultivation (A8P51_09170, accD1, pyrD, FD10_GL000649, and AY051_09565) for subsequent validation by quantitative real-time reverse transcription polymerase chain reaction (qRT-PCR). The relative fold expression of these identified proteins in the *luxS* mutant strain was significantly changed (all *P* < 0.01) compared to that in the wild-type strain in mono-cultivation and co-cultivation (Fig. 5). At the gene transcription level, the expression patterns of all 10 proteins corroborated the proteomic results.

## Discussion

Understanding the mechanism of QS regulation is indispensable to increasing our basic knowledge regarding environmental adaptation and improving the application of bacteria in the food industry^19^, especially when involving strategies for regulating QS in bacteriocin production. To illustrate the effects of *luxS* on bacteriocin production, we previously constructed a *luxS* mutant strain of *L. plantarum* KLDS1.0391 by homologous recombination and found that AI-2 activity of the *luxS* mutant strain was significantly lower (*P* < 0.01) than that of the wild-type strain during a 4–24-h growth period (unpublished data), regardless of mono-cultivation or co-cultivation with *L. helveticus* KLDS1.9207. This suggested that the *luxS* gene is necessary for the synthesis of AI-2 by *L. plantarum* KLDS1.0391. Moreover, we also found that the bacteriocin production and AI-2 activity in *L. plantarum* KLDS1.0391 are positively correlated^6^. In the present study, the bacteriocin production by and cell number of *L. plantarum* KLDS1.0391 were positively correlated during the logarithmic growth phase; this finding is consistent with that of cell population density-dependent regulation in QS^5^. Notably, the *luxS* gene had a large influence on cell number and bacteriocin production during co-cultivation but had no influence on these measures in mono-cultivation, as previously reported by Sztajer *et al*.^20^. This phenomenon revealed that the AI-2 signal export and reception/transduction pathways might differ between mono- and co-cultivation, resulting in bacteriocin production being ultimately sensitive to co- but not mono-cultivation. As shown in Fig. 3 and Supplementary Fig. S3, the results of the proteomic analyses are consistent with the above results. In particular, in response to *luxS* deletion in *L. plantarum* KLDS1.0391, the expression level of proteins involved in carbohydrate metabolism, amino acid metabolism, fatty acid synthesis and metabolism, and the two-component regulatory system changed (Tables 1 and 2).

In co-cultivation, 3-oxoacyl ACP reductase (FabG) and acetyl-CoA carboxylase carboxyl transferase subunit beta (accD), which are related to fatty acid synthesis, were at levels lower than the detection limit of MS in the *luxS* mutant strain, whereas these proteins were abundant in the wild-type strain. FabG is positively related to the synthesis of fatty acids and catalyses the conversion of 3-ketoacyl ACP to 3-hydroxyacyl ACP^21^. In turn, AccD can catalyse the conversion of acetyl-CoA to malonyl-CoA and is also the rate-limiting enzyme in fatty acid synthesis^22^. These results indicate that the *luxS* deletion in *L. plantarum* KLDS1.0391 decreased the synthesis of fatty acids in this bacterium, which constitute the main component of the cell membrane. Bacteria can regulate cell membrane fluidity by regulating the type and composition of fatty acids, thereby maintaining membrane stability and normal physiological function; they can also adapt to different stresses^23^, such as acid stress^24^, heat shock^25^, bile stress^26^, and osmotic stress^27^. Thus, our findings suggest that the growth and metabolism of the *luxS* mutant strain decreased because of the reduction in the amount of fatty acids synthesized, which would impair KLDS1.0391 cell membrane fluidity.

In comparison, the presence of the phosphotransferase system (PTS) in *L. plantarum* is related to sugar catabolism and may facilitate this activity^28^ as well as the growth of *L. plantarum*. The low expression of the glucitol/sorbitol-specific IIA component (PTS, srlB) suggested that deletion of *luxS* might affect the growth of *L. plantarum* KLDS1.0391. Furthermore, in the present study, the expression of aminotransferase (patA), which participates in amino acid synthesis and is positively correlated with the biosynthesis of amino acids, was below the MS detection limit in the *luxS* mutant strain. Notably, previous studies investigating the stimulation of bacteriocin production by organic nitrogen sources^29^ have shown that certain amino acids are necessary to synthesize the lanthionine ring (only in lantibiotics)^30^, that several amino acids (or peptides) act as enzymatic inducers^31^, and that normal bacterial growth has specific nutritional requirements^32^. Although these results are unclear, and the specific role of amino acids in bacteriocin production has not yet been satisfactorily identified, amino acids (or peptides) are assumed to be involved in bacteriocin biosynthesis. Thus, our finding of decreased patA expression might represent one of the causes of altered bacteriocin production in the absence of the *luxS* gene. However, the effect of amino acids on bacteriocin synthesis requires further investigation.

**Figure 6:**
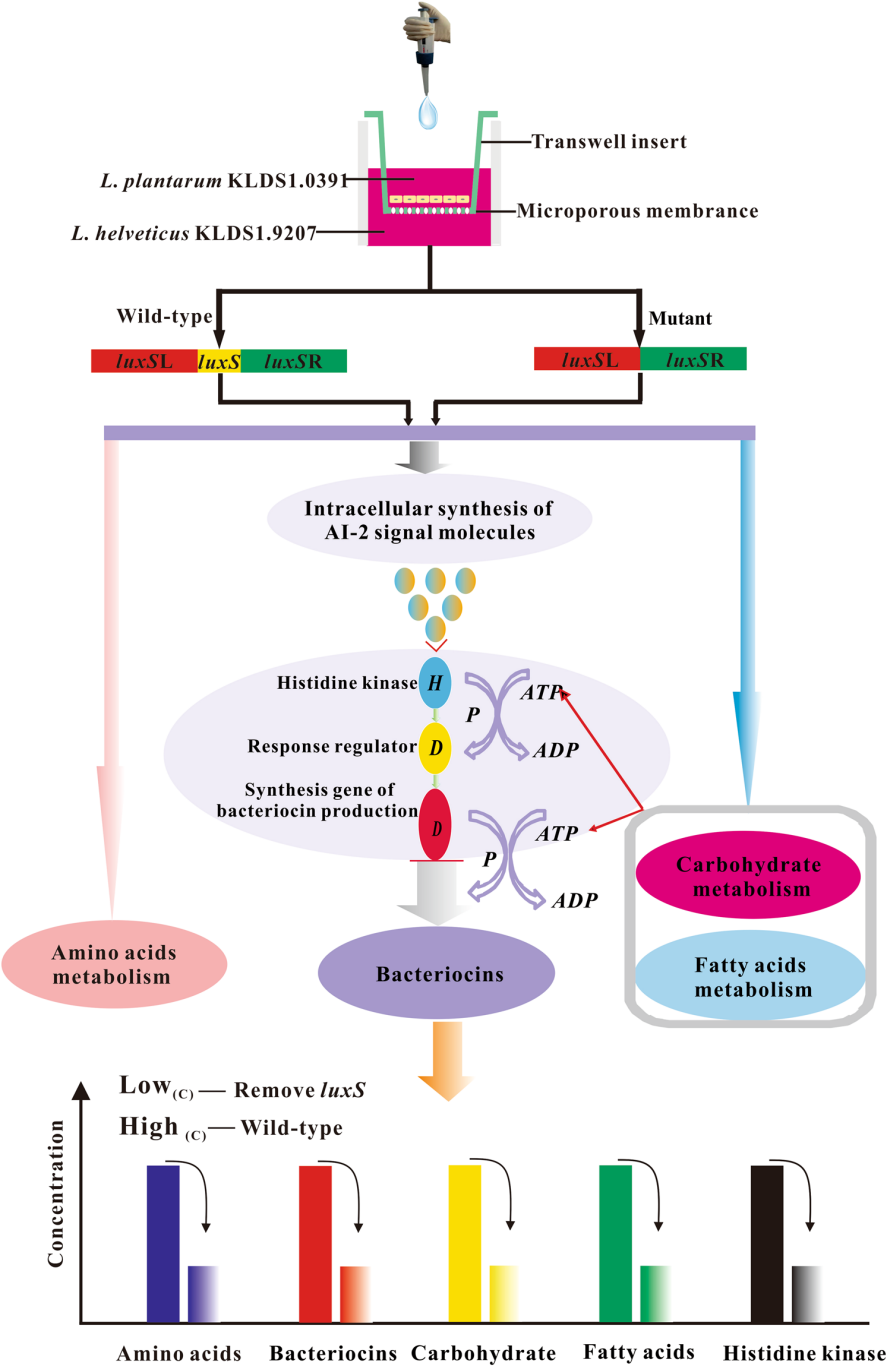
Possible mechanism of LuxS in bacteriocin biosynthesis by *L. plantarum* KLDS1.0391 in co-cultivation with *L. helveticus* KLDS1.9207. *luxS*L (1100 bp) and *luxS*R (1100 bp) represent the conserved left and right domains, respectively, of *luxS*.

The two-component regulatory systems that recognize AI-2 and oligopeptide signalling molecules in LAB are consistent with each other^33^. The histidine protein kinase serves as a membrane-localised receptor or sensor for signalling molecules and transfers this signal through a series of phosphorylation or dephosphorylation reactions to the cytoplasmic response regulator, which in turn binds DNA to activate transcription of the bacteriocin synthesis gene^33^. In the present study, the levels of sensor histidine kinases (AgrC, BlpH), which are necessary for the subsequent induction of bacteriocin production^34^, were lower than the detection limit of MS in the *luxS* mutant strain, whereas these were abundant in the wild-type strain (Table 2 and Fig. 3). Several previous studies^35,36^ found that co-cultivation of *L. acidophilus*, *L. sanfranciscensis* CB1, and *L. plantarum* DC400 could increase bacteriocin production and that energy-metabolism-related proteins are also upregulated. As the biosynthesis of bacteriocin is generally considered a process of energy dissipation, we speculated that bacteriocin production might be associated with energy production in the carbohydrate and fatty acid metabolic pathways, and that a large amount of energy would be utilised by the two-component system to further control bacteriocin synthesis. These phenomena may also decrease the bacteriocin production in the *luxS* mutant strain. In combination with the phenotypic results, the possible mechanism of *luxS* function in bacteriocin biosynthesis during co-cultivation with *L. helveticus* KLDS1.9207, as inferred by our findings, is shown in Fig. 6.

During mono-cultivation, in response to the deletion of the *luxS* gene, *L. plantarum* KLDS1.0391 decreased the levels of proteins involved in carbohydrate metabolism (e.g. pyruvate dehydrogenase E1 component alpha and beta subunits, pyruvate dehydrogenase E2 component, dihydrolipoamide dehydrogenase, and acetate kinase) and amino acid metabolism (e.g. dihydrolipoamide dehydrogenase and phosphoserine aminotransferase). Without such deletion, *L. plantarum* KLDS1.0391 increased the level of 3-oxoacyl-[acyl-carrier-protein] synthase III (FabH) and decreased the level of enoyl-[acyl-carrier protein] reductase II (FabK), which are involved in fatty acid synthesis. Pyruvate dehydrogenase E1 component alpha and beta subunits, as well as pyruvate dehydrogenase E2 component, are important constituent enzymes of the pyruvate dehydrogenase complex and are rate-limiting enzymes; they can also catalyse the irreversible oxidative decarboxylation of pyruvate to acetyl-CoA. The oxidation of sugars, the tricarboxylic acid cycle, and oxidative phosphorylation are related to acetyl-CoA, which plays an important role in mitochondrial respiratory chain energy metabolism^37^. The decrease in the level of pyruvate dehydrogenase E1 component alpha and beta subunits, as well as pyruvate dehydrogenase E2 component, showed that pyruvate was fermented to produce high amounts of lactic acid. Thus, *L. plantarum* KLDS1.0391 could accelerate the metabolic production of lactic acid in the absence of the *luxS* gene. In addition, the increase in FabH levels promoted fatty acid production, whereas the low level of FabK reduced fatty acid synthesis. These conflicting phenomena might lead to an unchanged metabolic capacity of *L. plantarum* KLDS1.0391 upon *luxS* gene knockout. In our previous study, we found that when the bacteriocin of *L. plantarum* KLDS 1.0391 was separated and purified, its molecular weight was approximately 2,180 Da, and the sequence of its five N-terminal amino acids was valine-proline-tyrosine-proline-glycine^14^. Therefore, we speculated that the decrease in levels of dihydrolipoamide dehydrogenase and phosphoserine aminotransferase observed in the present study regulated the metabolism of glycine, serine, threonine, valine, leucine, and isoleucine; such decreases might also reduce bacteriocin production.

In summary, the results indicated that AI-2 signal export and reception/transduction pathways differed between mono- and co-cultivation of *L. plantarum* KLDS1.0391. Moreover, the carbohydrate metabolism, amino acid metabolism, fatty acid metabolism, and two-component regulatory system pathways of *L. plantarum* KLDS1.0391 were altered when the *luxS* gene was deleted. Collectively, these pathways could influence the production of bacteriocin. In particular, carbohydrate and fatty acid metabolism pathways may provide energy for bacteriocin biosynthesis through QS. Future research will focus on the specific role of amino acids in the bacteriocin production by *L. plantarum* KLDS1.0391. These findings will provide a theoretical foundation for the effect of *luxS* on bacteriocin production using selective culture conditions.

## Methods

### Bacterial strains, media, and growth

*L. plantarum* KLDS1.0391 (wild-type strain and *luxS* mutant strain), *L. helveticus* KLDS1.9207, and *Bacillus subtilis* ATCC6633 were provided by the Dairy Industrial Culture Collection at the Key Laboratory of Dairy Science, China. *L. plantarum* KLDS1.0391 and *L. helveticus* KLDS1.9207 were grown in de Man, Rogosa, and Sharpe (MRS) broth at 37 °C. The *luxS* mutant strain of *L. plantarum* KLDS1.0391 contains chloramphenicol resistance genes, whereas the wild-type strain is sensitive to chloramphenicol. To prevent the *luxS* gene from recovering from the mutation and to restrain the growth of the wild-type strain, the *luxS* mutant strain was grown in MRS broth supplemented with chloramphenicol (10 μg/mL, Sigma, St. Louis, MO, USA). *B. subtilis* ATCC6633 was grown in beef extract-peptone broth at 37 °C. All strains were stored at −80 °C in 40% (v/v) glycerol and propagated twice at 37 °C for 16 h in their corresponding broth medium before use.

### Preparation of mono- and co-cultures

The tested extracts must be from a single strain to meet the requirements of proteomics analysis. All mono- and co-cultures were prepared as follows: 16-h-old cells of *L. plantarum* KLDS1.0391 wild-type and *luxS* mutant strains (approximately 10^9^ colony forming units (CFU)/mL) were inoculated (1%, v/v) separately into fresh MRS and grown at 30 °C for 6 h (mid-exponential phase of growth) to obtain mono-cultures. To identify the differential expression of proteins in a co-culture system, co-cultures were prepared in a way similar to that reported by Di Cagno *et al*.^12^. In the present study, a chamber was used to realize the exchange of small molecules under the co-culture system and ensure that the tested strains were pure. A structural model of the chamber is shown in Supplementary Fig. S1. Chambers containing fresh MRS broth were inoculated with 1% of an overnight culture of the wild-type or *luxS* mutant strain in the culture insert, followed by 0.5% of an overnight culture of the co-culture strain (i.e. approximately 10^8^ CFU/mL of *L. helveticus* KLDS1.9207 in the well); these chambers were then placed into an incubator at 30 °C for 6 h with gentle agitation (60 rpm). Co-cultivation was obtained from a double culture vessel apparatus separated by a 0.4-μm membrane filter (Millipore Isopore; Billerica, MA, USA). Each experiment was conducted in triplicate. Detection of membrane permeability and bacterial growth in the double chamber is shown in Supplementary S-1 (Supplementary Table S1 and Supplementary Fig. S2).

### Detection of live cell number and antibacterial activity

Co- and mono-cultivation were performed in MRS broth at 37 °C for 24 h, and samples of the culture were removed every 2 h to determine the live cell number by plate counting^6^. The antibacterial activities were analysed for each group using the modified ‘agar-well-diffusion-assay’ method^38^ with *B. subtilis* ATCC6633 as the indicator strain. The mono- and co-cultures of the wild-type strain were used as the positive controls for the assays of antibacterial activity. Inhibition zone diameter was used to indicate the antibacterial activity of bacteriocin^6,38^. *P* values < 0.05 were considered to indicate statistical significance.

### Extraction, quantification, and digestion of whole-cell proteins

Each culture was harvested (10,000 × *g* for 10 min at 4 °C), re-suspended in 500 μL SDT-lysis buffer (4% SDS, 100 mM Tris-HCl, 1 mM dithiothreitol, pH 7.6)^39^, boiled for 10 min, subjected to ultrasonic disruption (10 × 10 sec^−1^ pulses at 100 W, with 15 sec^−1^ intervals), and centrifuged at 14,000 × *g* for 30 min. After centrifugation, the supernatant was transferred to a new tube, and the proteins were quantified. The protein concentration was measured by the bicinchoninic acid (BCA) method. SDS-PAGE was performed to verify the protein quality and concentration. Digestion of protein (100 μg for each sample) was performed according to the filter-aided-sample-preparation procedure described by Wiśniewski *et al*.^39^ with modifications. The detailed protocol is described in Supplementary S-2.

### Liquid chromatography-electrospray ionization tandem MS analysis

The peptide mixture of each sample was separated on a high-performance liquid chromatography (HPLC) system (EASY-nLC 1000, Thermo Finnigan, San Jose, CA, USA). After HPLC separation, the peptides from all replicates were analysed using a Q-Exactive MS (Thermo Finnigan) for 120 min^40,41^. Notably, each sample was processed three times, and the MS experiments for each sample were performed in triplicate to avoid contingency of the date and assure data reliability. The liquid chromatographic conditions, elution gradient, and Q-Exactive MS requirements are described in Supplementary S-3.

### Data analysis

Maxquant software version 1.3.0.5 was used to analyse the original data obtained from the label-free quantification proteome study for peptide identification and protein quantification^42^. The MS experimental data were searched against Unipro-Lactobaci-55542 -20160803.fasta.database (Indexed sequence 55542, downloaded on 03-08-2016). The main parameters used for protein identification and quantitative analysis are presented in Supplementary Table S2. The abundances of the peptides occurring in all control and experimental groups were compared by one-way ANOVA, and the proteins listed were filtered based on the ratio >±2 and *P* value < 0.05^42^.

### Bioinformatics analysis

GO, KEGG pathway, and clustering enrichment analyses were performed. All the identified differential proteins were submitted to GO analysis using Blast2GO^43^. The identified differential protein sequences were blasted against the NCBI database (ncbi-blast-2.2.28 + -win32.exe), and the first 10 alignment sequences that satisfied E-value ≤ 1e^−3^ were reserved for subsequent analysis. The GO entries associated with the target protein set and the matched alignment sequences in step one were extracted using the Blast2GO Command Line (database version: go_201504.obo, download address: www.geneontology.org). KEGG Automatic Annotation Server software was used to classify the target protein sequences into KEGG orthology (KO) by comparison with the KEGG GENES database^44^, and the path information of the target protein sequences were obtained automatically in accordance with KO classification. An average linkage hierarchical clustering analysis of samples based on the Euclidean distance algorithm was implemented in Cluster3.0 (http://bonsai.hgc.jp/~mdehoon/software/cluster/software.htm) and the Java Treeview software (http://jtreeview.sourceforge.net).

### Validation by qRT-PCR

FabH1, ackA, Lp19_0357, AY051_10080, Lp19_2148, A8P51_09170, accD1, pyrD, FD10_GL000649, and AY051_09565 are involved in fatty acid metabolism, pyruvate metabolism, pyrimidine metabolism, amino acids, and the two-component regulatory system. Thus, they were chosen to determine the level of gene transcription by qRT-PCR and validate the results of proteomics. RNA isolation and distinct expression analysis of the 10 mRNAs were implemented by a modified version of the method described by Man *et al*.^6^. RNA isolation was implemented using an RNAprep Pure Bacteria Kit (Tiangen, Beijing, China), as recommended by the manufacturer. cDNA was synthesized using the PrimeScript® RT Reagent Kit (Takara, Dalian, China), as described by the manufacturer. qRT-PCR amplification and detection were performed using the ABI 7500 Fast Real-Time PCR System (Applied Biosystems, Foster City, CA, USA) with the Sybr® Premix Ex TaqTM (Takara), following the protocol supplied.

### Data availability

The authors declare that the data generated from the current study are available and have been deposited in iProX database (http://www.iprox.org/page/PDV014.html?projectld=IPX0001032000).

## Electronic supplementary material

Supplementary Information
