# Supplementary material for: Role of the luxS gene in bacteriocin biosynthesis by Lactobacillus plantarum KLDS1.0391: A proteomic analysis
Source: Sci Rep. 2017 Oct 24;7:13871. doi: 10.1038/s41598-017-13231-4 (PMC5654829; doi:10.1038/s41598-017-13231-4)
Supplement: Supplementary file 1 — Supplementary Information [file 41598_2017_13231_MOESM1_ESM.pdf]

Role of the *luxS* gene in bacteriocin biosynthesis of *Lactobacillus plantarum* KLDS1.0391: A proteomic analysis

Fang-Fang Jia [1, 2], Xue-Hui Pang [1, 2], De-Quan Zhu [1, 2, 3], Zong-Tao Zhu [1, 2], Si-Rui Sun [1, 2], Xiang-Chen Meng [1, 2, *]

[1] Key Laboratory of Dairy Science, Ministry of Education, Northeast Agricultural University, Harbin, 150030, China

[2] Synergetic Innovation Center of Food Safety and Nutrition, Northeast Agricultural University, Harbin 150030, China

[3] College of Life Sciences, Jiamusi University, Jiamusi, 154007, People's Republic of China

*Corresponding author. Tel.: +86-451-55191813. Fax: +86-451-55190340.
E-mail address: xchmeng@hotmail.com (X. C. Meng)

1. Detection of the double chamber effect in co-cultivation of *L. plantarum* KLDS1.0391 and *L. helveticus* KLDS1.9207

The membrane permeability was detected by the method described below. The results are shown in Table S1.

Detection of membrane permeability using double chamber				
	Group 1	Group 2	Group 3	Group 4
Upper chamber	*L. plantarum* KLDS1.0391	-	*L. helveticus* KLDS1.9207	-
Lower chamber	-	*L. plantarum* KLDS1.0391	-	*L. helveticus* KLDS1.9207
Each sample was incubated in MRS broth at 37 ℃ for 24 h with gentle agitation (60 rpm), and the culture was removed every 2 h for detecting the cell number of bacteria.				
Upper chamber	*	Determine the cell number of *L. plantarum* KLDS1.0391	*	Determine the cell number of *L. helveticus* KLDS1.9207
Lower chamber	Determine the cell number of *L. plantarum* KLDS1.0391	*	Determine the cell number of *L. helveticus* KLDS1.9207	*

"-" means that no strains are inoculated.
"*" means that the number of cell in this chamber is not determined.

The bacterial growth was detected by the following method. *L. plantarum* KLDS1.0391 was inoculated (1%) into the double chamber and triangular flask. Sample was incubated in MRS broth at 37 ℃ for 24 h with gentle agitation (60 rpm). The culture was removed every 2 h, and the number of viable cell of *L. plantarum* KLDS1.0391 with time changes in two culture modes was detected. The results are shown in Fig. S3.

2. SDS-PAGE was carried out on vertical electrophoresis apparatus (SE260, GE-Healthcare) at 15 mA and per gel using a 12% (w/w) poly-acrylamide separating gel overlaid with a 5% stacking gel. Samples were boiled for 5 min in SDS-PAGE 5×(5:1, v/v) Sample Loading Buffer (10% SDS, 0.5% BPB, 50% Glycerol, 500 mM DTT, 250 mM Tris-HCl, pH 6.8), and centrifuged at 14000 g for 10 min. The supernatant was transferred to a new tube. Each lane on the gel received 20 μg of protein for electrophoresis at 5 mA for 60 min. After electrophoresis, the

polypeptides were stained with Coomassie Brilliant Blue R250 and decolored with methanol/ acetic acid. Finally, the result of electrophoresis was analyzed by gel imaging system.

Filter-aided-sample-preparation procedure was described as followed. Briefly, each sample added DTT to a final concentration of 100 mM, boiled for 5min and cooled down to room temperature. Then, 200 μL UA buffer (8 M Urea, 150 mM Tris-HCl, pH 8.0) was added to remove the detergent, DTT and other low-molecular-weight components by repeated ultrafiltration (Microcon units, 30 kD) facilitated by centrifugation (14000 g, 15 min). Further, 100 μL 50 mM iodoacetamide (IAA, Bio-Rad) in UA buffer was added to prevent the reduced cysteine residues and stirred at 600 rmp for 1 min, and the samples were incubated for 30 min in total darkness and centrifuged at 14000 g for 10 min. The filter was washed twice with 100 μL UA buffer and 100 μL 25 mM NH_4HCO_3 buffer, respectively. Finally, the protein suspension was digested with 2 μg Trypsin (Promega) in 40 μL 25 mM NH_4HCO_3 buffer at 37 ℃ for 16-18 h, and the resulting peptides were collected as a filtrate. The peptides of each sample were first desalted with C18-SD Extraction Disk Cartridge and then estimated by UV light absorbance at 280 nm.

3. The detailed liquid chromatographic conditions and elution gradient were described below. A binary mobile phase, which was consisted of eluent A (0.1% formic acid-2% acetonitrile, v/v) and eluent B (0.1% formic acid-84% acetonitrile, v/v) with a gradient program of 0-45% B (0-100 min), 45-100% B (100-108 min) and 100% B (108-120 min), was used throughout the separation. During the sample injections, the capillary HPLC column (Thermo EASY column SC200 150 μm*100 mm, RP-C18) was re-equilibrated with 100% A. The peptide mixtures (2 μg) were loaded onto a RP-C18 column (SC001 traps 150 μm*20 mm) and the peptide separation was carried out in a RP-C18 column (SC200 150 μm*100 mm) at 400 nL/min as the gradient above.

The detailed Q-Exactive MS requirements were described below. The result of MS were acquired higher-energy collisional dissociation (HCD) fragmentation by choosing the positive ion and precursor ions range from 300 m/z to 1800 m/z. The resolution for survey MS scans was set at a value of 70,000 with a 200 m/z, and the HCD spectra were acquired a resolution of 17,500 with m/z of 200. The m/z of peptides and peptide fragments were collected by full scan and selected the twenty data-dependent MS/MS scans. The Normalized Collision Energy of 30 eV was used to activate precursor irons, and the Underfill ratio, which indicates the Mix percentage of the target value likely to be attained to the Max fill time, was defined as 0.1%.

Table S1 Detection effect of membrane permeability on *L. plantarum* KLDS1.0391 and *L. helveticus* KLDS1.9207

Time (h)	Group 1	Group 2	Group 3	Group 4
	L. plantarum KLDS1.0391		*L. helveticus* KLDS1.9207	
0	-	-	-	-
2	-	-	-	-
4	-	-	-	-
6	-	-	-	-
8	-	-	-	-
10	-	-	-	-
12	-	-	-	-
14	-	-	-	-
16	+	-	-	-
18	+	-	-	-
20	+	+	-	-
22	+	+	-	-
24	+	+	-	-

“-” means that no cells were detected, "+" means that cells were detected.

Group 1- *L. plantarum* KLDS1.0391 is inoculated into the upper chamber.

Group 2- *L. plantarum* KLDS1.0391 is inoculated into the lower chamber.

Group 3- *L. helveticus* KLDS1.9207 is inoculated into the upper chamber.

Group 4- *L. helveticus* KLDS1.9207 is inoculated into the lower chamber.

Table S2 Main parameters used for protein identification and quantitative analysis

Item	Value
Enzyme	Trypsin
Max Missed Cleavages	2
Main search	6 ppm
First search	20 ppm
MS/MS Tolerance	20 ppm
Fixed modifications	Carbamidomethyl (C)
Variable modifications	Oxidation (M), Acetyl (Protein N-term)
Database	Uniprot_Lactobaci_55542_20160803.fasta
Database pattern	reverse
Include contaminants	Ture
Peptide FDR	≤ 0.01
Protein FDR	≤ 0.01
Time window (match between runs)	2 min
Protein Quantification	Use razor and unique peptides
LFQ	Ture
LFQ min. ratio count	1

Supplementary Figure Captions

Fig. S1 The structural model of chamber.

Fig. S2 Growth curve of L. plantarum KLDS1.0391 under different culture conditions. Cell numbers are expressed as means $\pm$ SD (n=3).

Fig. S3 Quorum sensing pathway. The red represents the differential proteins in mono-cultivationon on the graphic pathway map.

Figure S1

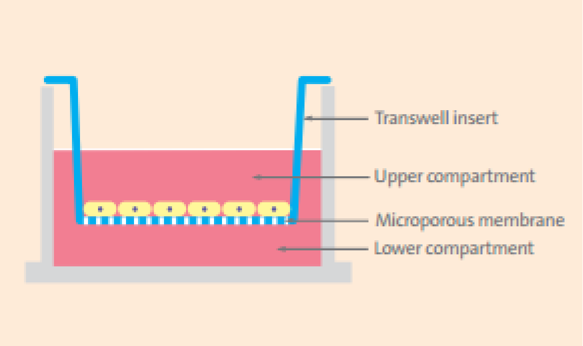

Transwell insert
Upper compartment
Microporous membrane
Lower compartment

Figure S2

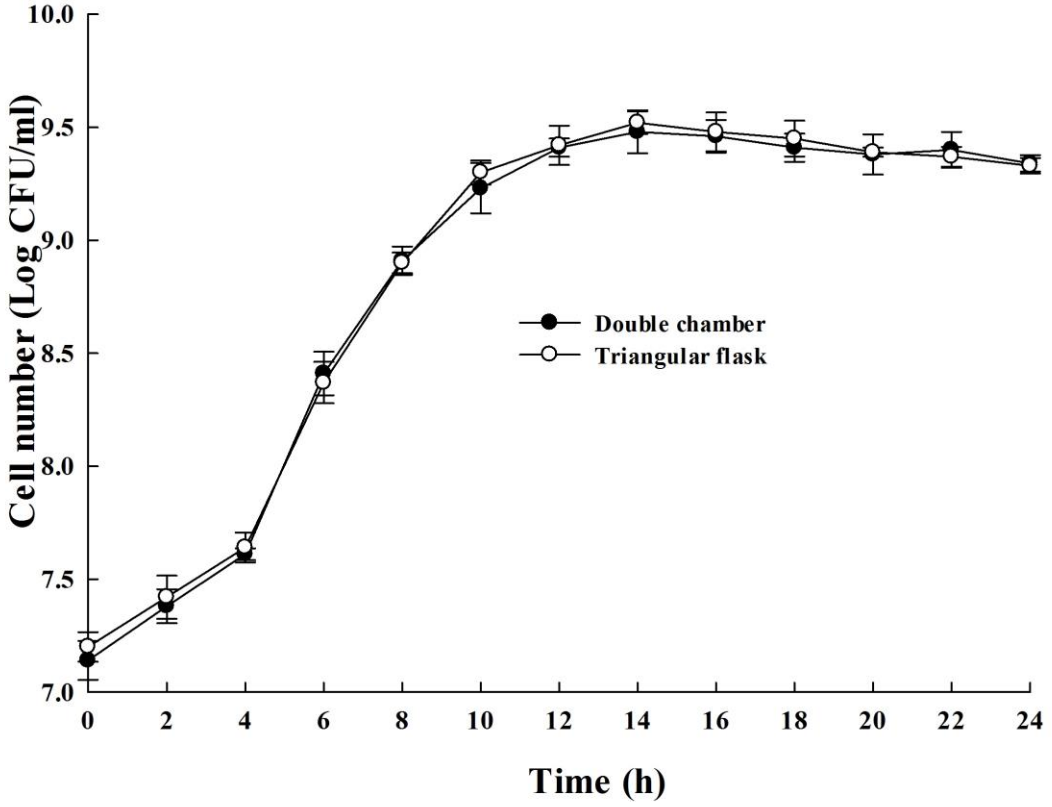

10.0
9.5
9.0
8.5
8.0
7.5
7.0
Cell number (Log CFU/ml)
0
2
4
6
8
10
12
14
16
18
20
22
24
Time (h)
Double chamber
Triangular flask

Figure S3

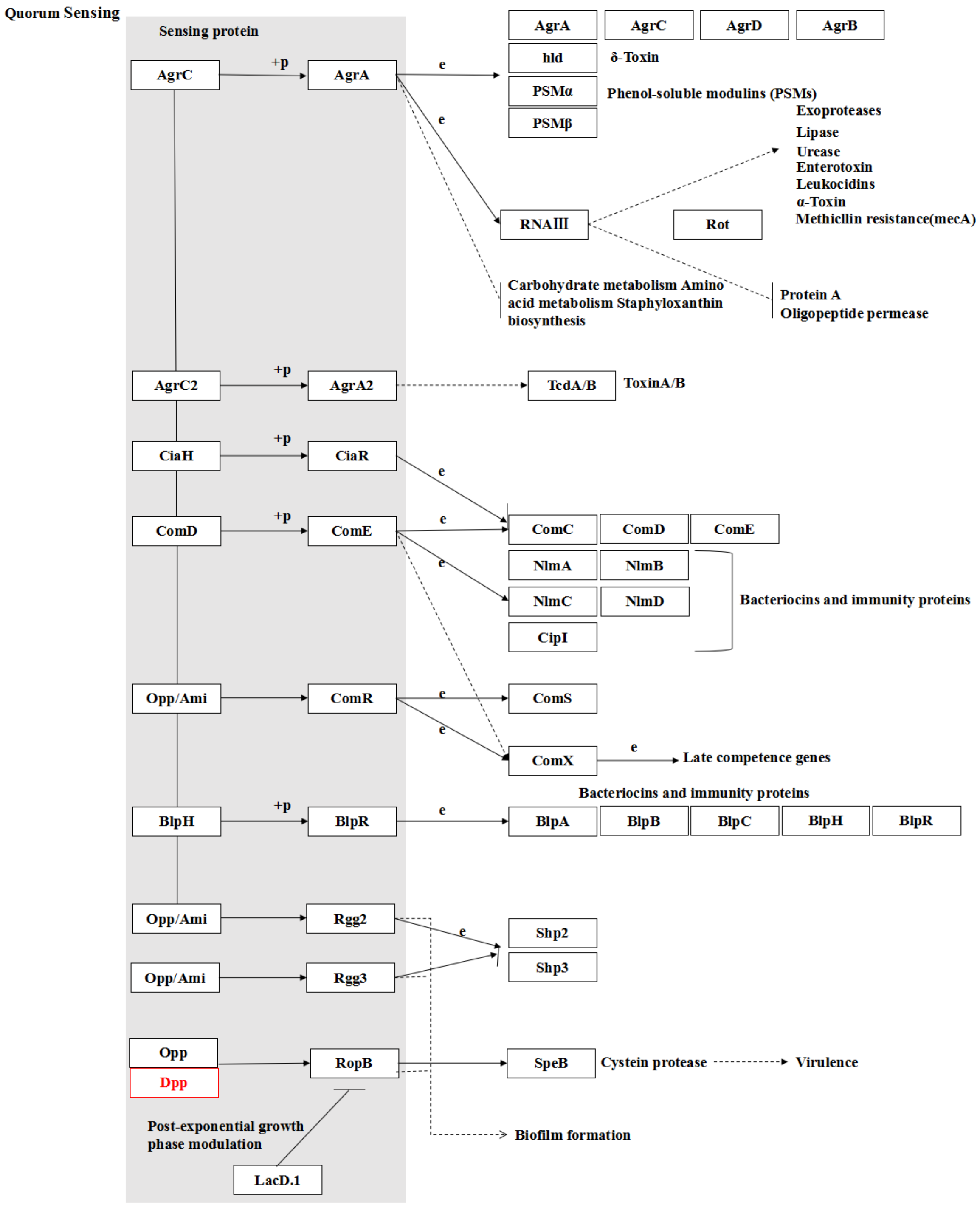

Quorum Sensing
Sensing protein
AgrC
+p
AgrA
e
AgrA
AgrC
AgrD
AgrB
hld
δ-Toxin
PSMα
Phenol-soluble modulins (PSMs)
PSMβ
Exoproteases
Lipase
Urease
Enterotoxin
Leukocidins
α-Toxin
Methicllin resistance(mecA)
RNAIII
Rot
Carbohydrate metabolism Amino acid metabolism Staphyloxanthin biosynthesis
Protein A
Oligopeptide permease
AgrC2
AgrA2
TcdA/B
ToxinA/B
CiaH
CiaR
ComD
ComE
ComC
ComD
ComE
NlmA
NlmB
NlmC
NlmD
CipI
Bacteriocins and immunity proteins
Opp/Ami
ComR
ComS
ComX
Late competence genes
Bacteriocins and immunity proteins
BlpH
BlpR
BlpA
BlpB
BlpC
BlpH
BlpR
Opp/Ami
Rgg2
Opp/Ami
Rgg3
Shp2
Shp3
Opp
Dpp
RopB
SpeB
Cystein protease
Virulence
Biofilm formation
Post-exponential growth phase modulation
LacD.1
